# Phonatory Dysfunction as a Preclinical Symptom of Huntington Disease

**DOI:** 10.1371/journal.pone.0113412

**Published:** 2014-11-19

**Authors:** Jan Rusz, Carsten Saft, Uwe Schlegel, Rainer Hoffman, Sabine Skodda

**Affiliations:** 1 Department of Circuit Theory, Faculty of Electrical Engineering, Czech Technical University in Prague, Prague, Czech Republic; 2 Department of Neurology and Centre of Clinical Neuroscience, First Faculty of Medicine, Charles University in Prague, Prague, Czech Republic; 3 Department of Neurology, Huntington-Centre NRW, St. Josef Hospital, Ruhr-University of Bochum, Bochum, Germany; 4 Department of Neurology, Knappschaftskrankenhaus, Ruhr-University of Bochum, Bochum, Germany; University of Iowa Carver College of Medicine, United States of America

## Abstract

**Purpose:**

Although dysphonia has been shown to be a common sign of Huntington disease (HD), the extent of phonatory dysfunction in gene positive premanifest HD individuals remains unknown. The aim of the current study was to explore the possible occurrence of phonatory abnormalities in prodromal HD.

**Method:**

Sustained vowel phonations were acquired from 28 premanifest HD individuals and 28 healthy controls of comparable age. Data were analysed acoustically for measures of several phonatory dimensions including airflow insufficiency, aperiodicity, irregular vibration of vocal folds, signal perturbations, increased noise, vocal tremor and articulation deficiency. A predictive model was built to find the best combination of acoustic features and estimate sensitivity/specificity for differentiation between premanifest HD subjects and controls. The extent of voice deficits according to a specific phonatory dimension was determined using statistical decision making theory. The results were correlated to global motor function, cognitive score, disease burden score and estimated years to disease onset.

**Results:**

Measures of aperiodicity and increased noise were able to significantly differentiate between premanifest HD individuals and controls (*p*<0.01). The combination of these aspects of dysphonia led to a sensitivity of 91.5% and specificity of 79.2% to correctly distinguish speakers with premanifest HD from healthy individuals. Some form of disrupted phonatory function was revealed in 68% of our premanifest HD subjects, where 18% had one affected phonatory dimension and 50% showed impairment of two or more dimensions. A relationship between pitch control and cognitive score was also observed (*r* = −0.50, *p* = 0.007).

**Conclusions:**

Phonatory abnormalities are detectable even the in premotor stages of HD. Speech investigation may have the potential to provide functional biomarkers of HD and could be included in future clinical trials and therapeutic interventions.

## Introduction

Huntington disease (HD) is an autosomal-dominantly inherited neurodegenerative disorder caused by an expansion in the number of CAG repeats in the IT15 gene [1], leading to widespread neuronal atrophy of both white and grey matter. Clinically, HD is associated with the progressive decline of both motor and cognitive function, as well as psychiatric disturbances. There is a growing body of evidence that progressive functional (e.g., tapping), cognitive and structural changes in the brain precede the clinical onset of HD by many years [2]–[7]. As predictive testing is available, there is great potential for the development and management of early treatment strategies in HD. Yet, the premanifest period is likely the most suitable period for the introduction of disease-modifying therapies in order to delay or even prevent symptomatic disease onset [8].

Motor manifestations of HD are generally characterized by involuntary movements termed chorea, which predominate in the initial and middle stages of the disease and are frequently later supplanted by rigidity and dystonia [9]. In addition, abnormalities of voluntary motor function such as problems with planning, initiation, tracing and termination of movements accompany chorea but may already be present in preclinical stages [10].

Speech impairment is a component of the common motor manifestations of HD, occurring in more than 90% of affected patients [11]. Typical signs of dysarthria in HD include voice dysfunction, articulation deficits, irregular loudness variation and abnormalities in speech timing [11]–[15]. As speech production requires the overall integrity of the central nervous system [16], one may hypothesize that subtle changes in speech may precede the clinical onset of HD. Accordingly, preliminary studies have reported greater incidence of low-frequency segments and decreased oral motor efficiency in subjects at risk for HD [17], [18]. While these findings appear promising, only one study sought patterns of subtle preclinical speech abnormalities in genetically proven premanifest HD (PreHD) [13]. This study focused mainly on aspects of speech timing and showed that speech performance tends to decrease with disease progression, however, comparison among groups revealed no significant differences between PreHD individuals and healthy controls [13]. In general, very little is known about different aspects of speech in the preclinical stages of HD and the potential of speech tests as a marker of clinical HD onset.

Considering that complex voice function has never been investigated in gene positive PreHD thus far, the purpose of the present study was to objectively identify phonatory changes that may occur in the premotor stages of HD by assessing a wide range and novel combination of dysphonia measurements. We hypothesized that PreHD patients should manifest subtle voice deficits during sustained vowel phonation, in agreement with previous observations suggesting that certain phonatory deficits are related to the subtle impairment of voluntary movements [14]. Furthermore, we investigated possible relationships between dysphonia features and clinical parameters such as global motor function, cognitive score, disease burden score and estimated years to disease onset to provide deeper insight into the pathophysiology of voice function in PreHD.

## Methods

### Subjects

A total of twenty-eight German native speakers (14 men, 14 women) with gene positive PreHD volunteered for the present study. Their ages ranged from 20 to 55 years (mean 37.1, standard deviation [SD] 9.3). Classification as PreHD was based on expert rater assessment of motor signs insufficient for a diagnosis of HD (Diagnostic Confidence Level, item 17 of the UHDRS Motor Assessment) [19], suggesting no substantial motor signs. Each PreHD participant underwent extensive neurological and neuropsychological evaluation. In addition to the motor UHDRS score, overall cognitive score (CS) including verbal fluency test, symbol digit modalities test, Stroop colour, Stroop word and Stroop interference subtests were calculated [19]. Subsequently, fine motor performance was assessed by a simple tapping test where higher motor performance leads to lower scores [20], as well as a more complex pegboard test where higher motor impairment leads to higher scores [21]. In addition, a disease burden score was computed using the formula (age × [CAG repeat - 35.5]) [22]. Years to onset of diagnostic motor manifestations were estimated on the basis of the survival analysis formula introduced by Langbehn et al. [23]. Clinical characteristics of the PreHD group are listed in Table 1.

**Table 1:** Clinical characteristics of PreHD subjects.

n = 28 (14 men)	Mean (SD)	Range
Age (years)	37.1 (9.3)	20–55
UHDRS motor score	2.2 (2.4)	0–8
Cognitive score	337 (44)	242–411
Tapping ζ	189 (23)	142–229
Pegboard ψ	4492 (805)	3469–7519
Disease burden score	251 (82)	116–413
Years to onset (years)	16.7 (8.2)	5–36

UHDRS  =  Unified Huntington's Disease Rating Scale.

ζ Number of taps within a time period of 32 seconds, average value of the dominant and non-dominant hand.

ψ Time period of peg insertion in 100 ms, average value of the dominant and non-dominant hand.

Twenty-eight healthy German speakers with no history of neurological and/or communication disorders were recruited as a control group (15 men, 13 women), of comparable age ranging from 24 to 55 years (mean 39.7, SD 9.3). No difference in age distribution was found between the PreHD and control groups (*p* = 0.31). The study was in compliance with the Helsinki Declaration and was approved by the Ethics Committee of Ruhr University Bochum. Written informed consent was obtained from each participant.

### Speech Data and Acoustic Analyses

Speech samples were digitally recorded in a quiet room using commercial audio software (WaveLab©, Steinberg, Hamburg, Germany) and a head-set microphone (Platronics Audio 550 DSP©, Platronics Inc., California, USA) positioned approximately 5 cm from the subject's lips. The audio data were sampled at 44.1 kHz with 16-bit resolution. During the recording, the participants performed various speaking tasks as a part of a larger protocol. For the current study, only recording of sustained phonation was used for further analyses, where each participant was instructed to take a deep breath and perform sustained phonation of the vowel/a/at a comfortable loudness and pitch, as constant and long as possible. All PreHD individuals performed the sustained vowel phonation twice with a high test-retest reliability (*r* = 0.71–0.94, *p*<0.001); the first trial of phonation was considered for final analysis.

For evaluation of voice deficits, acoustic analyses were preferred as they offer a non-invasive, valid and reliable method to precisely assess voice abnormalities. Recently, several dysphonia measurements were designed to examine HD-related voice dysfunction [14]. We further extended and elaborated this previous methodology, allowing the assessment of seven specific dimensions of phonatory dysfunction in PreHD individuals. To assess airflow insufficiency, we examined maximum phonation time (MPT) and time to first occurrence of voice break (FOVB) [14]. To investigate aperiodicity, we evaluated number of voice breaks (NVB), degree of pitch breaks (DPB) and degree of vocal arrest (DVA) [24]. With respect to irregular vibration of the vocal folds, we extracted pitch variability (F0 SD) and recurrence period density entropy (RPDE) [14], [25]. To examine signal perturbations, we investigated frequency instability (jitter) and amplitude instability (shimmer) [24]. To capture problems with increased noise, we calculated the harmonics-to-noise ratio (HNR) and detrended fluctuation analysis (DFA) [24], [25]. Considering vocal tremor, we applied frequency tremor intensity index (FTRI) and amplitude tremor intensity index (ATRI) [26]. To elucidate articulation deficiency, we proposed measures of articulator stability using Mel-frequency cepstral coefficients (MFCC) and delta MFCCs (ΔMFCC) [14]. All acoustic parameters were designed to be gender independent and to provide reliable automated assessment in clinical practice. Table 2 provides a detailed description of the measurements used in the present study.

**Table 2:** Overview of applied phonatory measurements.

Abbreviation		Description
**Airflow insufficiency**		
MPT (s)	Maximum phonation time	Aerodynamic efficiency of the vocal tract measured as the maximum duration of the
		prolonged vowel. This measure includes all voice breaks occurring during the entire
		vowel phonation.
FOVB (s)	First occurrence of voice	Maximum duration of the prolonged vowel until the first occurrence of the first voice break,
	break	present after at least 250 ms of modal phonation.
**Aperiodicity**		
NVB (−)	Number of voice breaks	Overall count of voice breaks. A voice break is defined as the distance between
		consecutive pulses longer than 1.25 divided by the bottom of the pitch range. The
		segment was defined as a voice break only if it occurred after at least 250 ms of modal
		phonation and 1 s preceding the termination of phonation. Voice breaks may be
		associated with both low frequency drop and vocal arrest.
DPB (%)	Degree of pitch breaks	The fraction of pitch frames marked as unvoiced. A frame was considered unvoiced if
		it had voicing strength below the voicing threshold of 0.45 (autocorrelation function).
		Silent periods were not considered in analyses.
DVA (%)	Degree of vocal arrests	The fraction of silent periods in the analysed voice signal.
**Irregular vibrations of vocal folds**		
F0 SD (st)	Standard deviation of	Variation in frequency of vocal fold vibration. The F0 sequence was converted to
	fundamental frequency (F0)	a semitone scale to avoid differences in gender.
RPDE (−)	Recurrence period density	Ability of the vocal folds to sustain simple vibration. RPDE quantifies the deviations
	entropy	from periodicity, representing the uncertainty in the measurement of the pitch period.
**Signal perturbations**		
Jitter (%)	Frequency perturbation	Extent of variation of the voice range. Jitter is defined as the variability of the
		fundamental frequency of speech from one cycle to the next.
Shimmer (%)	Amplitude perturbation	Extent of variation of expiratory flow. Shimmer is defined as the
		sequence of maximum extent of the signal amplitude within each vocal cycle.
**Increased noise**		
HNR (dB)	Harmonics-to-noise ratio	The amount of noise in the speech signal, mainly due to incomplete vocal fold closure.
		HNR is defined as the amplitude of noise relative to tonal components in speech.
DFA (−)	Detrended fluctuation	The extent of turbulent noise in the speech signal. DFA measures the stochastic
	analysis	self-similarity of the noise caused by turbulent airflow through the vocal folds.
**Vocal tremor**		
FTRI (%)	Frequency tremor intensity	Average ratio of the frequency magnitude of the most intense low-frequency modulating
	index	components to the total frequency magnitude of the analysed voice signal.
ATRI (%)	Amplitude tremor intensity	Average ratio of the amplitude of the most intense low-frequency amplitude modulating
	index	components to the total amplitude of the analyzed voice signal.
**Articulation deficiency**		
MFCC (−)	Mel-frequency cepstral	Vocal tract transfer function reflecting potential problems with subtle motion of the
	coefficients	articulators (jaw, tongue, lips). The MFCC parameter here was defined as the mean of
		the standard deviations of the 1st-12th MFCCs. It was designed to represent overall stability
		of individual vocal tract elements, as the individual MFCCs overlap the partitions of the
		frequency domain.
ΔMFCC (−)	Delta MFCCs	The ΔMFCC parameter represents a similar function as MFCC and was defined as the
		mean of the standard deviations of the 1st-12th delta MFCCs multiplied by 10.

### Statistics

Statistical analyses were performed in Matlab© (Mathworks, Massachusetts, USA). The Kolmogorov-Smirnov test was applied to test for normality and revealed that all acoustic parameters had a normal distribution with the exception of aperiodicity. To evaluate group differences, a two-sample t-test was used for normally distributed data while the nonparametric Mann-Whitney U-test was performed in other cases. The Pearson and Spearman correlations were applied to test for significant relationships of normally and non-normally distributed data, respectively. Post-hoc Bonferroni adjustment was applied to correct for the number of all tests performed according to specific phonatory dimension; the level of significance after adjustment was set to *p*<0.05. Effect sizes were determined with Coheńs *d*, with *d*>0.5 indicating a medium and *d*>0.8 indicating a large effect.

Wald decision theory was applied to compare the probability distributions between PreHD and healthy subjects and estimate the percentage of affected PreHD individuals according to the specific phonatory dimension [27]. Furthermore, classification experiment based on support vector machine (SVM) with Gaussian radial basis kernel was performed to find the combination of acoustic markers allowing the best discrimination (sensitivity/specificity) between PreHD individuals and controls. To validate reproducibility of SVM classifier, 4-fold cross-validation scheme was used where original data were randomly split into a training subset composed of 75% and testing subset composed of 25% of the data; this cross-validation process was repeated twenty times for each combination. Comprehensive details on classification procedure have been provided elsewhere [28], [29].

## Results

**Figure 1:**
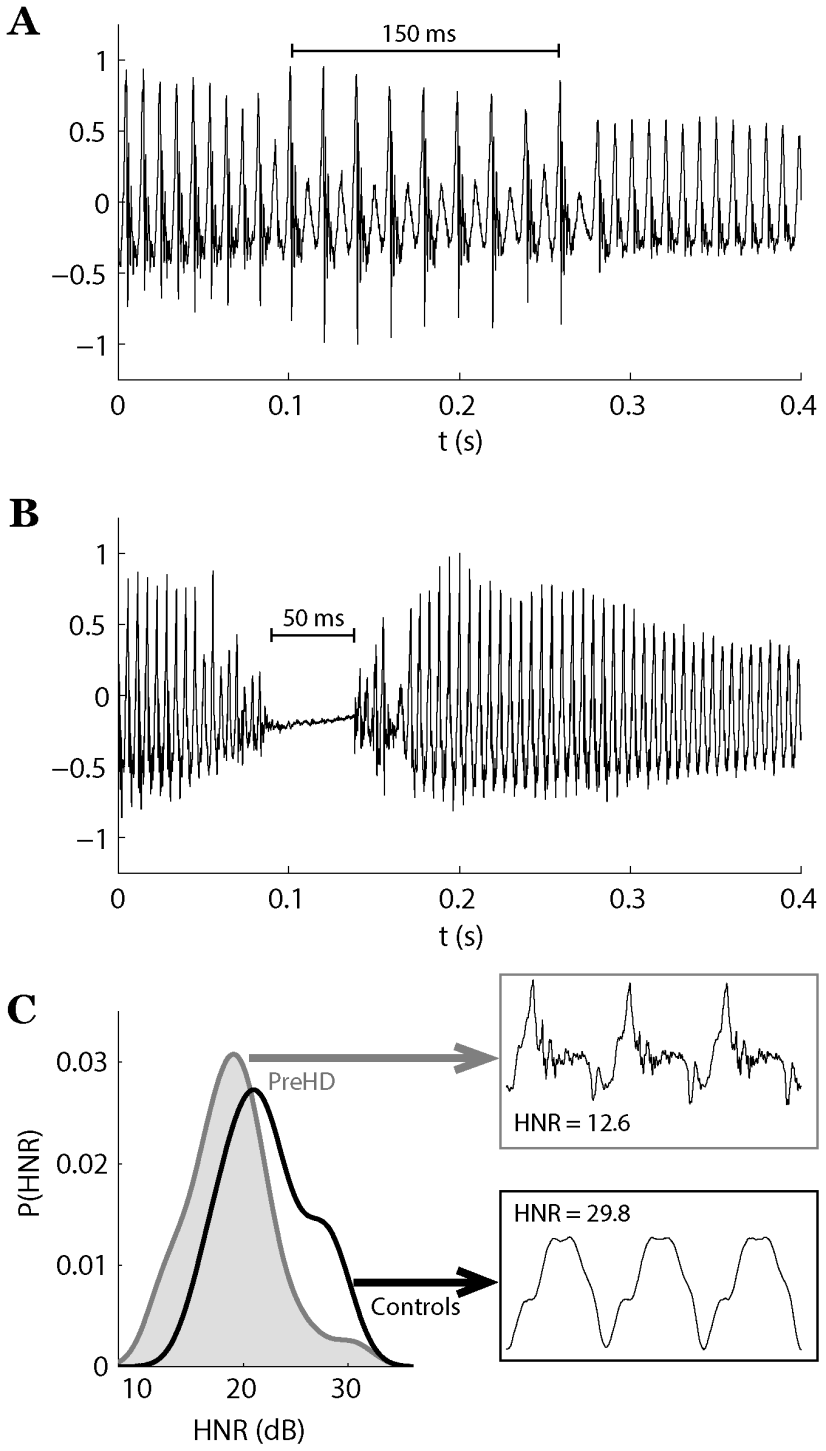
Most salient signs associated with phonatory dysfunction in PreHD individuals: (A) drop in the fundamental frequency to half of its original value over a short period of time; (B) short vocal arrest produced with no vibration of vocal folds; (C) probability density for ratio between harmonics and noise components in voice, with detail of three pitch periods for healthy and disordered voice.

Table 3 lists the numerical data and comparisons between the PreHD and control groups. From all investigated phonatory dimensions, the voice of PreHD individuals was mainly affected by the presence of aperiodicity and increased noise. In comparison to controls, the PreHD group showed significantly increased NVB (*p* = 0.007), DPB (*p* = 0.002), DFA (*p* = 0.0002) and decreased HNR (*p* = 0.007). Figure 1 provides visual guidance in recognizing the three main features identified (pitch break, vocal arrest and increased noise components) that are associated with early phonatory dysfunction in PreHD.

**Table 3:** Results of voice analyses in PreHD individuals and appropriate controls.

Parameter	Group				Effect size †
	PreHD		Controls		PreHD
	Mean (SD)	Range	Mean (SD)	Range	vs. controls
**Airflow insufficiency**					
MPT (s)	16.9 (7.6)	6.7–43.2	14.1 (4.3)	9.9–25.4	0.44
FOVB (s)	12.4 (6.8)	0.7–30.8	13.9 (4.4)	9.9–25.4	−0.26
**Aperiodicity**					
NVB (−)	1.11 (2.04)	0–7	0.04 (0.19)	0–1	0.74*
DPB (%)	0.63 (1.14)	0–4.50	0.09 (0.05)	0–0.24	0.77*
DVA (%)	0.09 (0.27)	0–1.14	0 (0)	0–0	0.46
**Irregular vibrations of vocal folds**					
F0 SD (st)	0.30 (0.16)	0.11–0.79	0.30 (0.13)	0.16–0.67	−0.02
RPDE (−)	0.28 (0.07)	0.14–0.41	0.26 (0.05)	0.18–0.38	0.48
**Signal perturbations**					
Jitter (%)	0.80 (0.55)	0.16–2.25	0.61 (0.29)	0.28–1.51	0.41
Shimmer (%)	3.69 (1.25)	1.03–8.05	3.18 (1.42)	1.47–8.39	0.38
**Increased noise**					
HNR (dB)	19.0 (4.1)	12.4–30.5	22.3 (4.0)	15.8–29.8	−0.81*
DFA (−)	0.64 (0.02)	0.59–0.66	0.62 (0.02)	0.58–0.66	1.15**
**Vocal tremor**					
FTRI (%)	0.37 (0.17)	0.07–0.73	0.42 (0.24)	0.07–1.05	−0.22
ATRI (%)	4.60 (3.39)	1.09–16.59	4.74 (2.27)	0.96–10.13	−0.05
**Articulation deficiency**					
MFCC (−)	0.43 (0.07)	0.32–0.61	0.41 (0.08)	0.31–0.65	0.26
ΔMFCC (−)	0.45 (0.06)	0.36–0.58	0.44 (0.07)	0.31–0.57	0.12

*p<0.01;

**p<0.001.

† Cohen's d: Effect size 0.8 is considered large, 0.5 is considered medium, and 0.2 is considered small.

MPT  =  maximum phonation time; FOVB  =  first occurrence of voice break; NVB  =  number of voice breaks; DPB  =  degree of pitch breaks; DVA  =  degree of vocal arrests; F0 SD  =  variability of fundamental frequency; RPDE  =  recurrence period density entropy; HNR  =  harmonics-to-noise ratio; DFA  =  detrended fluctuation analysis; FTRI  =  frequency tremor intensity index; ATRI  =  amplitude tremor intensity index; MFCC  =  Mel-frequency cepstral coefficients; ΔMFCC  =  delta Mel-frequency cepstral coefficients.

**Figure 2:**
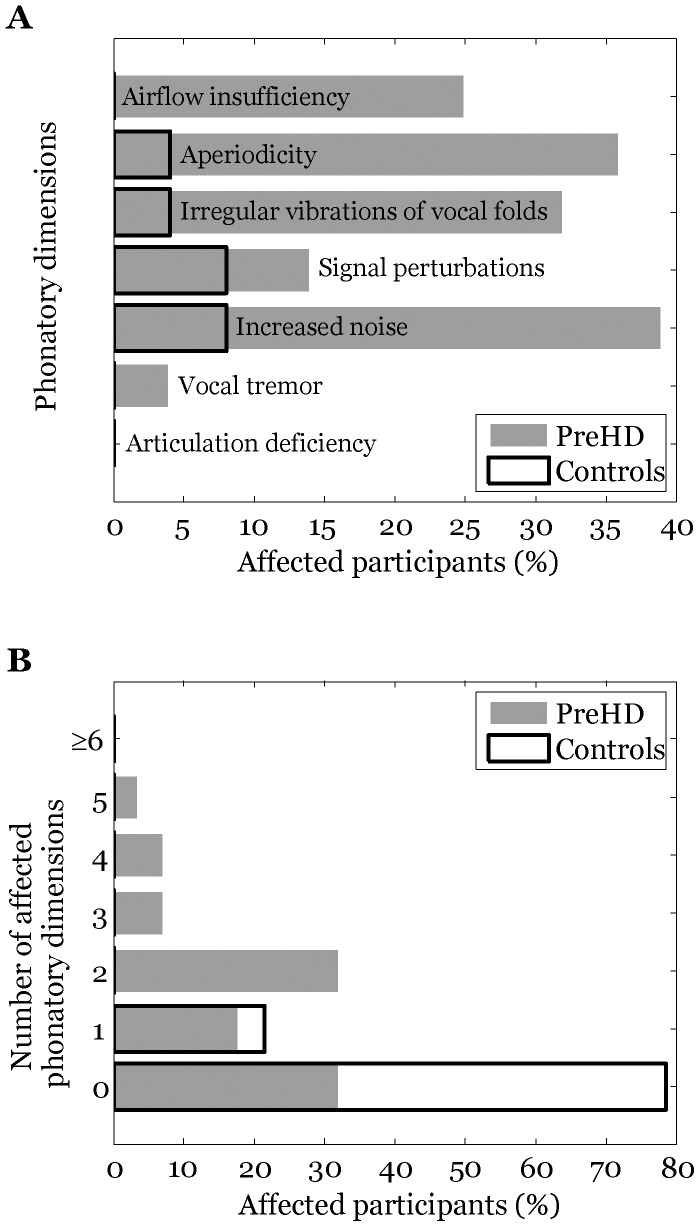
Results of voice analyses: (A) percentage of affected participants according to the specific phonatory dimension; (B) number of affected phonatory dimensions across participants.

Figure 2 highlights the percentage of affected subjects according to the specific phonatory dimension (Wald analysis). We observed that 19 PreHD subjects (68%) featured at least one affected phonatory dimension. One disrupted phonatory dimension was also observed in 6 controls (21%). Two or more affected phonatory dimensions were found in 14 PreHD subjects (50%), whereas no control speaker demonstrated more than one disrupted speech dimension. In the majority of individuals with two or more affected phonatory dimensions, at least one dimension was connected with aperiodicity or increased noise. In addition, using SVM classification model, we found that the combination of two aperiodicity measurements (DPB, DVA) and two noise measurements (HNR, DFA) leads to a sensitivity of 91.5±9.9% and specificity of 79.2±11.2% in discriminating PreHD from control participants.

We further observed significant relationships between cognitive score and both measures related to irregular vibration of the vocal folds (F0 SD: *r* = −0.43, *p* = 0.02; RPDE: *r* = −0.50, *p* = 0.007). No relationship was found between phonatory metrics and predicted years to disease onset or disease burden score, although there was a trend where PreHD subjects with some detected phonatory impairment were closer to the predicted age of disease onset (mean 16.1, SD 7.8 years) and showed higher disease burden scores (mean 258, SD 80) than those with a clear voice (years to onset: mean 17.8, SD 9.5 years; disease burden score: mean 238, SD 89). No other significant correlations were detected between phonatory and clinical parameters.

## Discussion

We detected phonatory dysfunction as a component of the subtle speech motor abnormalities occurring in the prodromal stages of HD. Measures of aperiodicity and increased noise were the most reliable in the prediction of PreHD group membership with 91.5% sensitivity and 79.2% specificity. Some form of disrupted phonatory function was revealed in 68% of our PreHD subjects while 18% had one affected phonatory dimension and 50% showed impairment in two or more dimensions. A relationship between pitch control and cognitive score was also observed. These findings provide new insight into the pathophysiology of speech disorders in premotor HD and may significantly contribute to existing assessment batteries.

Previous studies have shown that patients with manifest HD feature deficits at all phonatory levels, primarily reduced phonation time, aperiodicity, pitch fluctuations, increased noise and problems with articulator coordination [14], [30]. Only measures related to aperiodicity and increased noise were significantly aggravated than in controls in our PreHD group; we may thus hypothesize that most phonatory deficits observed in HD arise with the onset of worsening motor function. This hypothesis is further supported by our findings that PreHD speakers are free of markedly increased pitch variations as well as misplacement of articulators, which were previously found to be closely related to the extent of chorea [14]. In addition, respiratory problems associated with markedly reduced phonation time have been found to be pronounced with overall disease severity [30]. Nevertheless, increased addition of noise components in speech already seems to be present in the premotor stages of HD. Elevated noise is likely due to limited control of laryngeal muscles and incomplete vocal fold closure, resulting in inaccuracies in vibratory periods. In particular, similar pathophysiological mechanisms have also been suggested to be responsible for increased signal perturbations and irregular vocal fold vibrations, which were observed in several of our PreHD speakers.

Another manifestation of phonatory dysfunction in the prodromal stages of HD is the occurrence of voice breaks, which are associated with both short pitch drops and vocal arrests. Voice breaks have been hypothesized to be a consequence of hyper-adduction of the vocal folds and abnormal muscle tone [14], [30]. Conversely, a more reasonable explanation of voice breaks is motor impersistence, which is the inability to sustain certain simple voluntary motor actions such as maintaining a protruded tongue. Although short pitch drops may also be present in the vocalizations of the healthy population, their incidence was substantially increased in our PreHD group when compared to controls. Moreover, pitch drops in PreHD individuals frequently appeared in the first seconds of phonation, whereas they typically occur after a long period of phonation in healthy speakers [14]. Conversely, a few PreHD subjects produced short vocal arrests (about 50–300 ms in the present study), which can rarely appear in healthy vocalizations. Accordingly, vocal arrests seem to be a distinctive sign of HD although they have also been reported in certain cases of essential tremor [31].

Our current findings of early phonatory dysfunction are in agreement with data on impaired tongue force performance in a group of PreHD individuals approximately 16 years from expected disease onset [2], which is very similar to the predicted years to diagnosis in our PreHD group (17 years on average). Although deficits in tongue protrusion force coordination were sensitive enough to detect a motor phenotype early in prodromal HD stages [2], the sensitivity of both methods cannot be compared. On the other hand, when compared to controls, the tongue protrusion method reached a medium effect size [3], while our dysphonia measures showed large effect sizes. Indeed, phonatory tests may provide additional information as sustained phonation requires more sophisticated coordination of the vocal folds, jaw, tongue, palate and facial movements. Furthermore, the advantage of sustained phonation resides in fact that the subject's native language has no or a very small effect on dysphonia parameters, and therefore our findings in German language should be widely applicable to other languages.

However, contrary to previous research [2], we did not detect a relationship between the extent of phonatory dysfunction and disease burden score. One possible explanation is that some PreHD individuals already manifested certain phonatory abnormalities as a part of their habitual vocal behaviour. It is well known that the quality of speech differs among normal speakers and some isolated phonatory deficits were also seen in a few of our healthy controls. In addition, phonatory impairment in 94% of HD patients has recently been reported [14], suggesting that dysphonia do not develop in every individual in the course of premotor HD stages. Therefore, future longitudinal studies are needed to confirm and further elaborate our findings and to show the sensitivity of phonatory measurements as a potential marker of disease onset and progression.

The pathophysiological mechanism responsible for the phonatory deficits in PreHD revealed in the present study still has to be elucidated. Dysphonia with increased noise may be part of various types of motor speech disorders and therefore is likely a rather non-specific marker of neuronal dysfunction [16]. Conversely, recent imaging data have shown involvement of the putamen during the sustained phonation task [32], and putaminal gray matter loss had been detected in PreHD individuals several years before the estimated manifestation of the disease [2]. Notably, we also observed correlations between the cognitive score and measures of irregular vocal fold vibrations, which may be related to dysfunction of the frontostraital pathways [2].

Although the healthy control group was without previous history of neurological or communication disorders, one potential limitation of the current study is that controls did not undergo rigorous neurological evaluation including UHDRS motor score, cognitive score, tapping, and pegboard. On the other hand, possible subclinical neurological symptoms of any kind that could have been missed in our control group would rather lead to an attenuation of the differences between preHD and controls.

In conclusion, we provide the first objective assessment of a wide range of voice dimensions in prodromal HD, revealing a significant pattern of dysphonia with effect sizes comparable to those found in other studies investigating PreHD subjects. As speech tests are easy to perform, non-invasive and inexpensive, and acoustic analysis of speech can provide objective and quantifiable measures, speech investigations may have the potential to provide functional biomarkers of HD and could be included in future clinical trials and therapeutic interventions.
